# Monitoring the clinical practice of palliative sedation (PALSED) in patients with advanced cancer: an international, multicentre, non-experimental prospective observational study protocol

**DOI:** 10.1186/s12904-022-01125-w

**Published:** 2023-01-28

**Authors:** Maaike Rijpstra, Kris Vissers, Carlos Centeno, Johan Menten, Lukas Radbruch, Sebastiano Mercadante, Michael Van der Elst, Claudio Adile, Maria Arantzamendi, Evelien Kuip, Sheila Payne, Nancy Preston, Jeroen Hasselaar

**Affiliations:** 1 Department of Primary and Community Care, Radboud University Medical Centre, Nijmegen, the Netherlands; 2 Department of Anaesthesiology, Pain and Palliative Medicine, Radboud University Medical Center, Nijmegen, The Netherlands; 3 Departement of Palliative Medicine, Clínica Universidad de Navarra, Pamplona, Spain; 4 Department of Oncology, Laboratory of Experimental Radiotherapy, Katholieke Universiteit Leuven, Leuven, Belgium; 5 Department of Palliative Medicine, Universitaetsklinikum Bonn, Bonn, Germany; 6 Main Regional Center of Supportive/Palliative Care, La Maddalena Cancer Center, Palermo, Italy; 7 International Observatory On End of Life Care, Division of Health Research, Faculty of Health and Medicine, Lancaster University, Lancaster, UK; 8 Institute for Culture and Society-ATLANTES, Universidad de Navarra, Pamplona, Spain; 9 IdISNA, Instituto de Investigación Sanitaria de Navarra, Pamplona, Spain

**Keywords:** Palliative sedation, Observational study, End of life care, Advanced Cancer, Monitoring

## Abstract

**Background:**

Palliative sedation involves the intentional lowering of consciousness at the end of life. It can be initiated to relieve a patient’s burden caused by refractory symptoms at the end of life. The impact of palliative sedation needs to be clinically monitored to adjust the proper dose and regimen of sedative medication to ensure that patients are at ease and comfortable at the end of their lives. Although there is consensus among health care professionals and within guidelines that efficacy of palliative sedation needs to be closely monitored, there is no agreement about how, when, and by whom, this monitoring should be performed. The aim of this study is to evaluate the effects of palliative sedation by measuring the discomfort levels and sedation/agitation levels of the patients at regular timepoints. In addition, the clinical trajectories of those patients receiving palliative sedation will be monitored and recorded.

**Methods:**

The study is an international prospective non-experimental observational multicentre study. Patients are recruited from in-patient palliative care settings in Belgium, Germany, Italy, Spain and the Netherlands. Adult patients with advanced cancer are monitored by using proxy observations of discomfort (DS-DAT) and depth of sedation/agitation levels (RASS-PAL) during palliative sedation. After the palliative sedation period, the care for the specific participant case is evaluated by one of the attending health care professionals and one relative via a questionnaire.

**Discussion:**

This study will be the first international prospective multicenter study evaluating the clinical practice of palliative sedation including observations of discomfort levels and levels of sedation. It will provide valuable information about the practice of palliative sedation in European countries in terminally ill cancer patients. Results from this study will facilitate the formulation of recommendations for clinical practice on how to improve monitoring and comfort in patients receiving palliative sedation.

**Trial registration:**

This study is registered at Clinicaltrials.gov since January 22, 2021, registration number: NCT04719702.

## Background

An increase in cancer incidence together with a longer survival time for patients with advanced cancer is observed. Increasing treatment possibilities are available but are accompanied with progressively more morbidity. Therefore, the number of patients in need for end-of-life care is likely to increase [1]. Patients with advanced cancer may experience symptoms due to metastatic disease, but also due to treatment related side effects that significantly influence their quality of life such as pain, delirium or dyspnea [2]. For most terminally ill patients, these symptoms can be controlled towards the end of life with conventional treatment strategies. However, in some patients the symptom burden persists despite optimal treatments, resulting in ‘refractory symptoms’ [3]. For these patients with intolerable suffering and a limited life expectancy, palliative sedation may be indicated. This involves the intentional lowering of consciousness at the end of life, to relieve a patient’s perception of suffering [4]. The intensity of palliative sedation can vary in terms of the level of sedation, which can be mild, intermediate or deep, and the type of the sedation which can be intermittent or continuous [5]. Reported incidence of palliative sedation varies greatly between countries and between individual care settings. In some European countries the use of palliative sedation, mainly continuous deep sedation, has been studied [6, 7]. In these studies, the reported use of continuous deep sedation in patients with a palliative care need in different palliative care settings varies between 2.5% (Denmark) and 18.3% (The Netherlands) [7].

Most guidelines recommend that the level of palliative sedation should be proportional to the intended clinical effect of symptom relief [8]. Previous studies demonstrated a large variation of clinical evaluation methodologies to monitor the clinical efficacy of palliative sedation [9, 10]. Additionally, close monitoring of discomfort during palliative sedation is rare [11, 12].

Moreover, major differences between study protocols and characteristics occur, for instance in patient characteristics, definition of palliative sedation, and its indication to start. Consequently, reported outcomes and associated treatment goals differ between these studies which prevent the generalization of outcomes and decisions about the effective use of palliative sedation in clinical practice [10].

To understand the observed clinical outcomes of palliative sedation in patients with cancer suffering from refractory symptoms, it is important to relate these to the reasons to start palliative sedation, the prescribed drugs and dosages and the specific clinical context where they are measured. Moreover, differences across various palliative care settings and European countries in both clinical and ethical aspects should be included in the interpretation of the results [13]. So far, multinational clinical research for this purpose is lacking. Hence, there is an urgent need for internationally and prospectively observational standardized research on palliative sedation.

To study the clinical practice and outcomes of palliative sedation from different perspectives, we conducted a prospective multicenter non-experimental observational study in cancer patients from palliative care settings, in five European countries.

### The primary objective of this study is

1. To evaluate the effects of palliative sedation on advanced cancer patient’s comfort levels in hospices, palliative care units and hospital wards in five European countries.

### Secondary objectives

1. To evaluate the effects of palliative sedation on patient’s sedation levels in hospices, palliative care units and hospital wards in five European countries.
2. To describe the current clinical practice of palliative sedation in hospices, palliative care units and hospital wards in five European countries.
3. To describe the evaluations of the relatives and health care professionals with the palliative sedation given in the patient cases.

## Methods

### Design

In this clinical observational study, participants with advanced and untreatable cancer will be followed prospectively after informed consent given prior to the start of the decision-making process for end-of-life care until death. Before and during the palliative sedation in a patient, prospective measures and clinical observations will be performed to evaluate the sedation process. After the patients’ death, one of the attending health care professionals and one of the relatives will receive a questionnaire to evaluate the palliative sedation and care in the specific patient case.

### Participating settings

This prospective study will be conducted in five European countries: Belgium; Germany; Italy; Spain and the Netherlands. Participants are recruited from in-patient palliative care settings: hospital wards; palliative care units and hospices. In each country, we are aiming to recruit 30 participants receiving palliative sedation.

### Sample size calculation

In the study by van Deijck and colleagues [11] a mean difference of 4 points was found in comfort levels measured with the DS-DAT before and after the start of palliative sedation.

Because the present study will aim to include a wider range of settings and with more variability in forms of palliative sedation, therefore the effects may be smaller. Estimating a smaller effect size of 2.4 points on the DS-DAT, using an alpha of 0.05 and 0.80 power in a two-sided, paired t-test, with a standard deviation of 6.2, and correcting for clustering due to the multiple international centre settings (using an ICC of 0.05 and pre-assumed design effect of 1.2) we will need to include 110 sedated patients. To anticipate at missing data and dropout rate during the study we aim to include 150 sedated patients in total.

### Study population

All adult patients with a diagnosis of advanced cancer and a limited life expectancy, as assessed by the attending health care professionals, are eligible for inclusion. Additionally, intractable distress caused by one or more symptoms during the hospitalization needs to be present or expected, according to the health care team. Lastly, patients need to be able to give informed consent (see Table 1).

For the included patients, data are collected only during admission to one of the participating settings. For patients who are discharged alive or die without palliative sedation, data collection and study participation ends.

In each patient case that will be monitored during palliative sedation, the following persons will be invited to participate by completing an evaluation questionnaire after the palliative sedation period: 1) one of the health care professionals (physician or nurse) involved in the initiation of palliative sedation; 2) one of the relatives, who takes care of, and supports, the patient for most of the time. This relative may not necessarily be a family member.

**Table 1:** Inclusion- and exclusion criteria

**Inclusion criteria:** Patients are eligible for inclusion If they are;	•18 years or older; •diagnosed with advanced cancer; •having a limited life expectancy; •suffering from intractable distress caused by one or more refractory symptoms or this can be expected to develop during the hospitalization; •having the possibility that palliative sedation will be initiated during the admission •able to give informed consent.
**Exclusion criteria:** Patients are excluded for participation in the study if;	•a potential participant is unable to speak and read in the native language of the participating country.

### Recruitment

All consecutively admitted patients in the participating settings will be screened for eligibility by a health care professional from the local participating setting. Eligible patients and their relative(s) will be informed by the responsible health care team about this study as soon as possible after admission. This team defines in each particular case what is the best timing to ask for informed consent.

Screening and recruitment for the study started May 2021, and will be in progress for 24 months.

### Study procedures

Potential participants receive the information letter via one of the responsible health care professionals in the participating setting. When a potential participant has had sufficient time to consider and indicate that he/she would like to know more about participating, the local researcher is notified. One of the local researchers will visit the patient to explain the study, answer questions and discuss participation. Preferably, the informed consent procedure is performed by one of the local researchers independent of the patient’s care team. In addition, at each clinical centre, there is a local principal investigator that serves as a primary contact point for the study or to perform the informed consent procedure. Informed consent is requested for obtaining data about the patient’s treatment trajectory following admission.

### Study outcomes

The main outcome of this study is the proxy measurement of change in discomfort levels of patients before and during the period they receive palliative sedation.

As a secondary outcome, proxy measurement of agitation/sedation levels will be measured at the same time points as the discomfort levels, to evaluate a correlation in the course of both measurements.

Other outcomes in this study are clinical aspects in the process of decision-making about palliative sedation; administered type, doses of sedative medication and adverse effects as reported in the patients notes and in the case report forms. Besides, one of the health care professionals completes a questionnaire regarding the overall evaluation of palliative sedation in the specific patient case. Lastly, one of the relatives is asked to rate how satisfied he/she was with the care for the patient during the palliative sedation period.

### Data collection

**Fig. 1:**
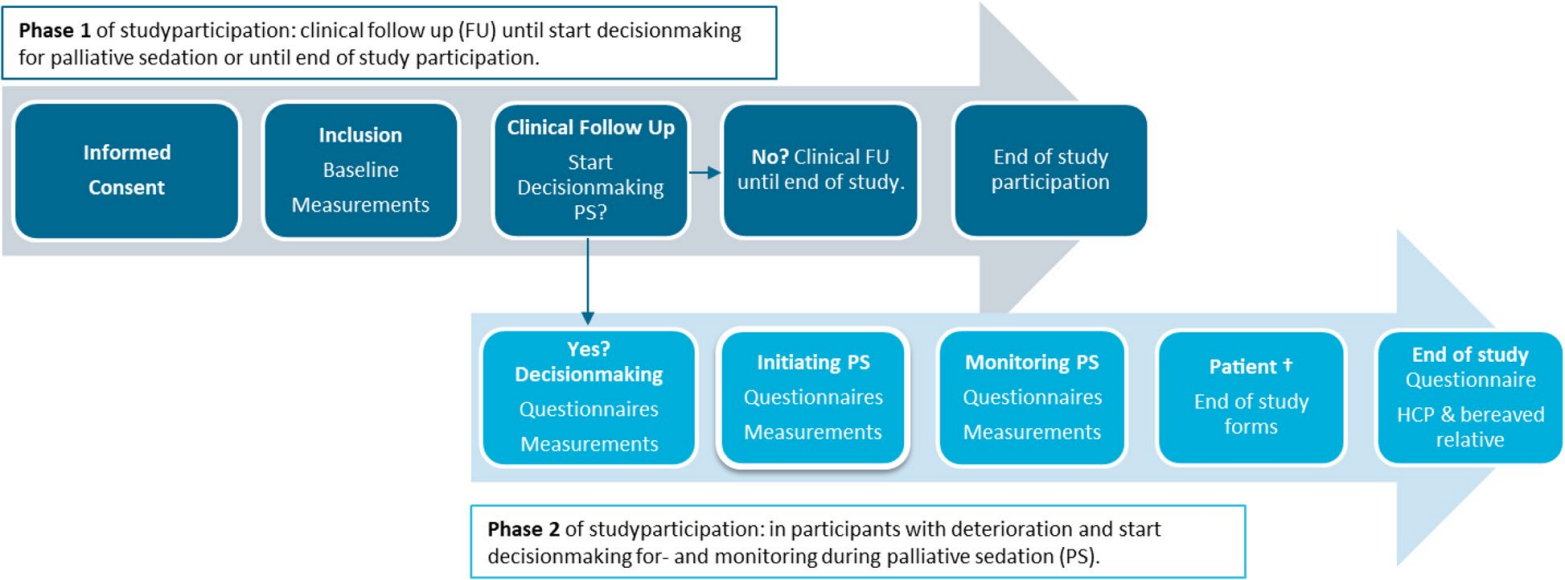
Study flow phase 1 and phase 2. All study participants start in phase 1 of the study, and participants in which the decision making is started regarding palliative sedation enter phase 2 of the study, all participants reaching the end of study due withdrawal consent, discharge or decease

Data collection is planned to use a two-stage approach (See Fig. 1). When informed consent is gained, baseline information for all patients is collected for phase 1. Then study measurements are paused and participants are clinically followed by the health care team.

Only those patients in which the process for palliative sedation is initiated will advance to phase 2 of the study. In this phase, extra questionnaires and measurements are completed by the health care professionals at the bedside, regarding the decision-making process of palliative sedation, baseline discomfort levels, the use of medication and other clinical aspects of palliative sedation.

To gather data about the study parameters we predominantly use data that is normally collected in clinical practice at the participating settings. This data is gathered from the available patient data management system (PDMS), and directly registered into the electronic case report form. Required study data regarding baseline measurements and all palliative sedation measurements, not regularly registered in the PDMS, are collected by study questionnaires or measured by additional scales.

**Fig. 2:**
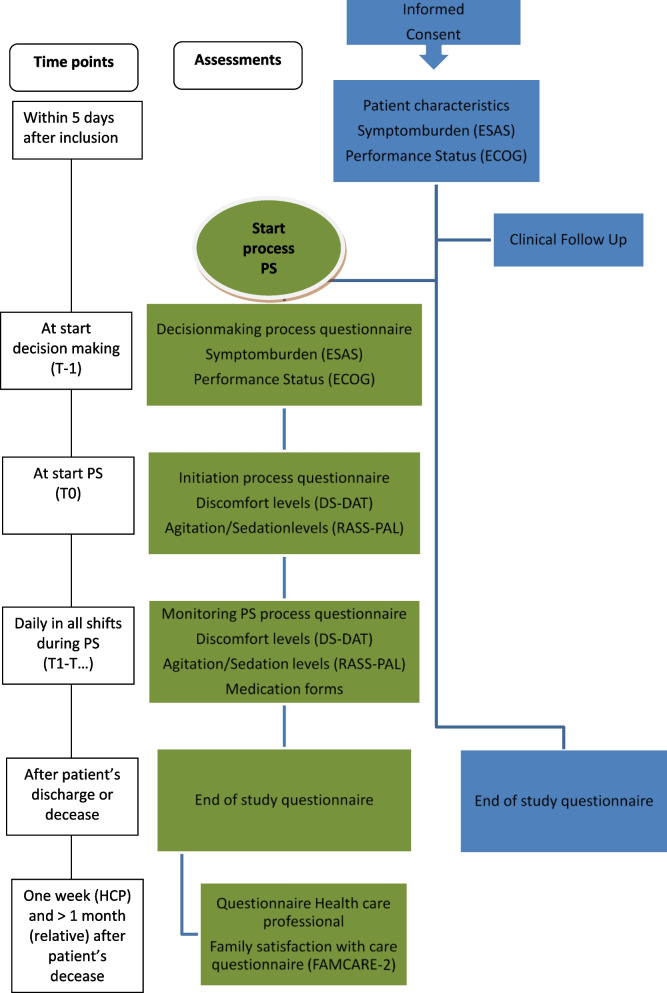
Study timeline and assessments. The measurements and questionnaires are completed at different time points during the trajectories of the study participants, For the participants who start with palliative sedation more assessments are planned

Assessment of variables is planned at different time points during the trajectories of the study participants, and contains more measurements for the participants who start with palliative sedation (See Fig. 2).

### Baseline measurements

#### Symptom burden

The Edmonton Symptom Assessment System (ESAS) is an assessment scale designed to assess the symptom burden of the patient. It contains 9 items, scored on a visual analogue scale [14]. As baseline measurement, all participants are asked to complete the Edmonton Symptom Assessment System (ESAS) after inclusion in the study. A second measurement will be done before initiation of palliative sedation by the patient themselves if possible or as a proxy measurement by the health care professional.

#### Performance Status

The Eastern Cooperative Oncology Group (ECOG) Performance Status is a scale that uses standard criteria to measure the impact of the disease on patient’s daily living abilities. The scale describes a patient’s level of functioning in terms of their ability to care for themself, conduct daily activities, and physical ability [15]. The ECOG performance score is completed by one of the attending physicians or clinical researchers as a baseline measurement in this study.

### Outcome measurements during palliative sedation

#### Monitoring comfort

The Discomfort Scale-Dementia of Alzheimer Type (DS-DAT) is a validated instrument to assess discomfort in patients with severe dementia by observing patients’ behavior over 5 minutes of observation. Participating health care teams were trained online and at bedside how to use the DS-DAT tool. The tool covers nine categories: noisy breathing, negative vocalizations, content facial expression, sad facial expression, frightened facial expression, frown, relaxed body language, tense body language, and fidgeting. Items are scored by indicating behaviors that are present/absent with a resulting range of scores of 0 to 3 per item and a total score from 0 to 27. Higher scores represent higher amount of discomfort [16].

In our study the DS-DAT is measured 8 hours or less before the start of palliative sedation, within 6 hours after the start, and then twice daily during the period palliative sedation is provided by one of the attending health care professionals.

#### Depth of sedation

The Richmond Agitation Distress Scale-modified version for Palliative care (RASS-PAL) is an observational instrument assessing levels of sedation and agitation that requires no patient input with scores ranging from + 4 (overtly combative patient) to − 5 (no arousal by either voice or physical stimulation) [17].

The depth of sedation as assessed with the RASS-PAL will be measured at the same time points as the discomfort measurements.

#### Reported signs and symptoms during palliative sedation

The presence of the following clinical signs and/or symptoms will be registered in the daily monitoring forms: breathing difficulties (dyspnea, rattle); respiratory secretions; (drug induced) delirium; paradoxical reaction/agitation; patients regaining consciousness; and other clinical observations.

#### Evaluation of the palliative sedation trajectories by the attending health care professionals

The attending health care professionals will be asked about their evaluation of the palliative sedation period for the specific patient. Daily rating of the effectiveness of palliative sedation at patient’s comfort level will be scored on a Likert Scale ranging from excellent to very poor.

1 week after the participants death, a questionnaire will be sent to one of the health care professionals, with three questions 1) about their personal rating of agreement to start palliative sedation in this patient, scored on a Likert scale ranging from 1 strongly disagree to 5 strongly agree, 2) the overall effect of the palliative sedation at the comfort levels, and 3) the quality of dying in the particular patient’s case, both scored on a 5-points Likert scale ranging from 1 very poor to 5 excellent.

#### Evaluation of the palliative sedation trajectories by one of the relatives

Two different methods will be used to evaluate the experiences of the relatives with the palliative sedation. First, a daily reporting by the attending health care professionals in the case report forms of expressed concerns and/or complaints of relatives during the palliative sedation period.

Furthermore, at least 1 month after the participant’s death, a structured questionnaire will be sent to one of the relatives. This family satisfaction with care questionnaire (FAMCARE-2) contains 17 items about the care received, which are scored on a Likert scale between 1 very unsatisfied to 5 very satisfied [18].

#### Medication used during the palliative sedation period

Changes in type-, dose- and route of administered sedative(s) and other medication during the palliative sedation period will be recorded daily on monitoring forms from the start of palliative sedation until the time of death.

This medication list contains information about: 1) Changes in dosages of sedative medication which is recorded in numbers and units (mg or mg/hr.); 2) Changes in the sort of sedative medication used for palliative sedation which are recorded with the medication name; 3) Changes in the route of administration of sedative medication. All changes will be recorded with the accompanying date and time.

#### Clinical aspects of decision making

During the decision-making process about palliative sedation, the involved health care professionals will complete questionnaires regarding aspects related to this process. The questionnaires contain questions regarding the initiation and timing of the decision-making and the involvement of various stakeholders (patient, relatives, health care professionals) in different stages of the process.

### Statistical analysis

Statistical analysis will be performed using SPSS, version 25 (SPSS, 2017, Inc. Chicago, IL) and R software, version 3.6.0 (The R Foundation for Statistical Computing, Vienna, Austria).

Missing data will be explored, reason for missing data will be reported and, when missing at random, they will be imputed with multiple regression imputation [19].

We will use a multilevel approach for analysis of changes in the repeated measures of discomfort and levels of sedation during palliative sedation. We will use these measurements as dependent variables and time and baseline measurements as independent variables. Secondary analyses will include possible confounding or effect modifying factors such as age, gender, country and setting.

Descriptive analysis will be performed, using proportions for categorical variables and mean with standard deviations for continuous variables, or median with interquartile range for data with skewed distribution.

Differences between settings will be tested through a Chi-square test for categorical variables (Fischer’s exact test in case of cell frequencies < 5), Kruskal-Wallis test for ordinal variables, and ANOVA for continuous variables.

The association between variables such as age, gender, country, setting, primary diagnosis, performance status and amount of symptom burden, with dosages of medication, levels of discomfort and length of sedation as continuous dependent variables, will be analysed in multivariable analyses.

### Dissemination

Dissemination of our findings will be presented at relevant conferences, meetings and through peer-reviewed journals, and is expected at the end of 2023. Within the EU funded project Palliative Sedation other dissemination plans are foreseen, such as an online education programme. More information about all dissemination activities can be found at the project website (www.palliativesedation.eu).

## Discussion

This study will explore the clinical practice of palliative sedation at the end of life in different palliative care settings in five European countries, and will study similarities and differences in indication, practice and specific outcomes of palliative sedation in patients with advanced cancer suffering from refractory symptoms.

### Strengths

The PALSED study is the first international multicenter study in which the palliative sedation trajectories of advanced cancer patients are observed in a standardized manner and with the main focus on the levels of discomfort in relation to the levels of sedation. Use of the same measurements in an international and multicenter context across different in-patient palliative care settings will provide valid results and may increase generalizability. Because participants can be included in the study earlier on during their palliative trajectory, in most participants the process of palliative sedation can be followed, from decision-making until the patients’ death and even afterwards by the involvement of relatives.

The use of observational measurement scales by experienced palliative care specialists, makes their evaluation more standardized and interpretable. In all settings and countries, the same questionnaires and clinical items will be observed and evaluated in a clear standardized manner. Since the main goal of palliative sedation is patient comfort, we presume that the level of symptom relief, and not unconsciousness as such, is used to guide titration of medication. Therefore, change in perceived discomfort levels could be an appropriate measurement for effectiveness, possible in combination with depth of sedation. When achievement of adequate sedation can be monitored in a patient by levels of discomfort this might result in a more tailored and proportional approach of the palliative sedation in the specific patient.

### Limitations

During preparatory meetings it became apparent that there were differences between countries and settings in the way information about palliative sedation was given. Differences in wording, timing and recipients of the given information. To stay as close as possible to daily practice, differences in the patient information sheets between the countries are respected in this study as are differences in timing of the informed consents.

Patients with rapid deterioration where the decision to start palliative sedation is very sudden may be missed for inclusion, since the time for informed consent procedures is limited and they need to give informed consent themselves. When these patients are included, missing data can be expected for measurements when the clinical situation requires a rapid response and time for study measurements are limited.

The absence of a validated scale for measuring comfort in patients with palliative sedation is a limitation, but discomfort in non-communicative patients at the end of life has been studied before. In patients receiving continuous palliative sedation, van Deijck and colleagues used discomfort as the primary outcome [11] which was also measured with the DS-DAT [15]. They concluded that the face validity of the DS-DAT for monitoring discomfort in palliative sedated patients appears to be good, as the items of the scale correspond to the current clinical assessment and the recommendations of some guidelines of measuring discomfort by facial expressions and body movements. Soto-Rubio et al. studied the initial psychometric properties of a discomfort observation scale, containing the same items as the DS-DAT, in a cohort of patients with palliative sedation and concluded that it had an adequate model fit [12].

In an earlier version of the protocol, we had envisioned that independent observers would complete the measurements of discomfort levels [20]. However, due to the start of the study recruitment during the COVID-19 pandemic, we were faced with limited access to the in-patient settings and participants. Therefore, we decided to train health care professionals working at the bedside in the measurements of DS-DAT and RASS-PAL. This can be seen as a limitation of the study because there is no longer an independent measurement. On the other hand, using the observations of experienced caregivers and collecting them in a uniform way can also be a strength which might support the daily clinical practice of monitoring simultaneously.

## Data Availability

The datasets generated during and analysed during the current study will be made publicly available. To permit the widest re-use of data as possible, the non-sensitive data will be made available as open access in DANS EASY. Before submitting the data in DANS, the Radboud University will check the data quality.

## Abbreviations

DS-DAT: Discomfort Scale--Dementia of Alzheimer Type
RASS-PAL: Richmond Agitation Distress Scale-modified version for Palliative care
ESAS: Edmonton Symptom Assessment System
ECOG PS: Eastern Cooperative Oncology Group Performance Status
